# Understanding the effect of angina on general and dimensions of psychological distress: findings from understanding society

**DOI:** 10.3389/fpsyt.2023.1119562

**Published:** 2023-05-25

**Authors:** Weixi Kang

**Affiliations:** Department of Brain Sciences, Imperial College London, London, United Kingdom

**Keywords:** angina, psychological distress, GHQ-12, depression, anxiety, anhedonia, social dysfunction, loss of confidence

## Abstract

**Background:**

The current study aimed to examine how the general and dimensions of psychological distress are affected by angina.

**Methods:**

First, a confirmatory factor analysis (CFA) was used to produce the three-factor solution of the GHQ-12. Second, a predictive normative modeling approach to predict the expected scores for 1,081 people with angina based on a model trained on demographics from 8,821 age and sex-matched people without angina. Finally, one-sample *t*-tests were used to determine the differences between the actual psychological distress scores and expected psychological distress scores in participants with angina.

**Results:**

There were three underlying structures of the GHQ-12 labeled as GHQ-12A (social dysfunction & anhedonia), GHQ-12B (depression & anxiety), and GHQ-12C (loss of confidence). Moreover, participants with angina had more psychological distress as indicated by the GHQ-12 summary score (Cohen’s *d* = 0.31), GHQ-12A (Cohen’s *d* = 0.34), GHQ-12B (Cohen’s *d* = 0.21), and GHQ-12C (Cohen’s *d* = 0.20) comparing to controls.

**Conclusion:**

The current study implies that GHQ-12 is a valid measure of psychological distress in people with angina, and there is a need to consider the dimensions of psychological distress in angina rather than solely focusing on certain dimensions of psychological distress such as depression or anxiety issues in people with angina. Clinicians should come up with interventions to reduce psychological distress in people with angina which can then lead to better outcomes.

## 1. Introduction

Psychological distress involves an alteration in an individual’s emotional state because of one’s inability to respond to life pressures or unmet demands (1). Psychological distress generally can be expressed in various forms such as depression, anxiety, and anhedonia. Moreover, these negative symptoms tend to overlap, which leads to the cooccurrence of various symtoms (2, 3). Drapeau et al. (2) estimated that the general population’s prevalence of psychological distress ranges from 5% to 27%. The 12-item general health survey (GHQ-12) has been examined in the literature as a reliable measure of psychological distress (11, 13, 23, 31, 41, 42) and has good specificity, reliability, and sensitivity (4, 5). The GHQ-12’s dimensionality has been a topic of controversy, with advocates for the unidimensional scale citing the high correlation between the identified factors (6, 7) and many others supporting the use of the 3-factor model, including GHQ-12A, GHQ-12B, and GHQ-12C (6–13). Recent studies have demonstrated that the imposition of a simple structure may artificially generate high correlations between modeled factors [e.g., (14)]. Thus, as suggested by Griffith and Jones (15), “taking these correlations as justification for unidimensionality risks a self-fulfilling prophecy of simplicity begetting simplicity.” In light of the ongoing debates regarding the structure of the GHQ-12, the present study investigates both the unidimensional and multidimensional models of the measure.

Angina is a prevalent symptom of coronary artery disease; however, it may also arise from other non-coronary artery related conditions, such as respiratory disease, valvular disease, anemia, and hyperthyroidism. These conditions can predict the occurrence rates of cardiovascular diseases and myocardial infarction independently of age. Moreover, alterations in pain sensitivity and stimuli *via* the vagal nerve fibers that are shared with the heart can result in non-cardiac chest pain resembling angina, which can be induced by gastrointestinal disorders. A meta-analysis revealed a wide prevalence range of angina, ranging from 0.7% to 15% (16–19).

It is important to understand psychological distress in angina given poorer cardiovascular disease outcome in people with higher psychological distress (20). Research has demonstrated that individuals who experience higher levels of psychological distress tend to have a greater number of comorbidities and physical disabilities (21), which may indicate the psychological distress is associated with severe disease, which in turn leads to poorer outcomes (22). Other studies have also looked at the possible biological mechanisms between psychological distress and poor outcome (23, 24), and suggested that secondary prevention may explain such a relationship. For instance, people with higher levels of psychological distress may be unlikely to participate in cardiac rehabilitation programs and change their lifestyles such as quitting smoking and increasing physical activities (25, 26), which then leads to adverse outcomes.

Indeed, there are some studies that have looked at the association between psychological distress and angina [e.g., (1, 21, 27–32)]. However, previous studies have primarily examined small clinical samples and focused on specific psychological distress such as depression and anxiety. There is limited research on the impact of angina on general psychological distress and different dimensions of psychological distress in population-based studies. Therefore, this study aims to investigate the relationship between angina and dimensions of psychological distress. Specifically, the study hypothesizes that there are three underlying factor structures of the GHQ-12: GHQ-12A (social dysfunction and anhedonia), GHQ-12B (depression and anxiety), and GHQ-12C (loss of confidence). Additionally, the study predicts that participants with angina will experience higher levels of both general psychological distress and dimensions of psychological distress.

## 2. Methods

### 2.1. Data

The study utilized data from the first wave of Understanding Society: the UK Household Longitudinal Study (UKHLS), which collects yearly information from a representative sample of UK households since 1991 under the name of The British Household Panel Study (BHPS; University of Essex, 2022). All participants provided informed consent prior to the study, which was conducted between 2009 and 2010. To ensure comparability, age and sex-matched healthy controls were selected, and those with incomplete data on the variables of interest were excluded from the analysis. The final sample consisted of 1,081 participants with angina and 8,821 age and sex-matched controls without a clinical diagnosis of angina. Table 1 provides descriptive statistics.

**Table 1:** The demographic characteristics of healthy controls and people with angina.

	Healthy controls		People with angina	
	Mean	S.D.	Mean	S.D.
Age	67.83	8.04	67.84	12.11
Income (monthly)	1233.89	1408.77	1059.56	839.12
	*N*	%	*N*	%
**Sex**				
Male	4,883	55.36	603	55.78
Female	3,938	44.64	478	44.22
**Education**				
Below college	7,242	82.10	937	86.68
College	1,579	17.90	144	13.32
**Marital status**				
Single	3,053	34.61	459	42.46
Married	5,768	65.39	622	57.54
**Residence**				
Urban	6,252	70.88	813	75.21
Rural	2,569	29.12	268	24.79

### 2.2. Measures

#### 2.2.1. Angina

The validity of self-reported angina has been appraised [e.g., (33)]. Participants answered the question “Has a doctor or other health professional ever told you that you have any of these conditions? Angina.” to indicate if they have been clinically diagnosed with angina.

#### 2.2.2. Psychological distress

The level of psychological distress was evaluated in this study using the GHQ-12, a unidimensional questionnaire that consists of 12 items designed to measure psychological distress (34). The Likert method was utilized to score the responses, ranging from 0 to 3, with 0 indicating “Not at all” and 3 indicating “Much more than usual.” A cumulative score of all 12 items was calculated to reflect the overall level of psychological distress, with higher scores indicating greater distress. For the factor analysis, responses on the GHQ-12 were rescored from 1 to 4, with 1 representing “Not at all” and 4 representing “Much more than usual.”

#### 2.2.3. Demographic variables

Demographic variables in the linear models include age, sex, income (monthly), education, marital status, and residence.

### 2.3. Analysis

#### 2.3.1. Factor model

In this study, a confirmatory factor analysis (CFA) using oblique rotation was conducted in MATLAB 2018a. The CFA was performed based on a pre-specified three-factor structure, with each factor representing a distinct dimension of mental health: GHQ-12A (social dysfunction and anhedonia, consisting of 6 items), GHQ-12B (depression and anxiety, consisting of 4 items), and GHQ-12C (loss of confidence, consisting of 2 items). The GHQ-12 summary score and factor scores were standardized with a mean of 0 and a standard deviation of 1 for further analysis.

#### 2.3.2. Predictive normative modeling

The study utilized a three-step predictive normative modeling approach. Firstly, four generalized linear models were trained using non-clinically diagnosed angina participants’ demographics as predictors and GHQ-12 summary score, GHQ-12A, GHQ-12B, and GHQ-12C as predicted variables. Secondly, expected scores were generated using the demographic information of angina patients as predictors in the generalized linear models. Lastly, one-sample *t*-tests were conducted to compare actual and expected scores in individuals with angina. This method was preferred over paired-sample *t*-tests since it allowed for demographic covariate control.

## 3. Results

The results of the factor analysis produced three distinct factors that were labeled as GHQ-12A, GHQ-12B, and GHQ-12C. GHQ-12A consisted of six items related to social dysfunction and anhedonia, GHQ-12B included four items related to depression and anxiety, and GHQ-12C consisted of two items related to loss of confidence. The specific loadings for each item can be found in Table 2.

**Table 2:** The factor loadings for the three-factor structure of the GHQ-12.

GHQ-12 Items	GHQ-12A (social dysfunction & anhedonia; 6 items)	GHQ-12B (depression & anxiety; 4 items)	GHQ-12C (loss of confidence; 2 items)
Concentration	**0.57**	0.20	−0.11
Loss of sleep	0.01	**0.68**	0.02
Playing a useful role	**0.61**	−0.17	0.13
Constantly under strain	**0.74**	−0.13	−0.02
Problem overcoming difficulties	−0.03	**0.86**	−0.08
Unhappy or depressed	0.08	**0.50**	0.20
Losing confidence	**0.57**	0.23	−0.12
Believe worthless	**0.69**	−0.05	0.04
General happiness	0.01	**0.53**	0.34
Capable of making decisions	0.01	0.17	**0.72**
Ability to face problems	0.09	−0.01	**0.73**
Enjoy day-to-day activities	**0.49**	0.12	0.12

The current study found a main effect of age [*F*(1, 8,814) = 23.88, *p* < 0.01], sex [*F*(1, 8,814) = 25.12, *p* < 0.001], monthly income [*F*(1, 8,814) = 16.36, *p* < 0.001], highest educational qualification [*F*(1, 8,814) = 12.39, *p* < 0.001], legal marital status [*F*(1, 8,814) = 57.09, *p* < 0.001] and residence [*F*(1, 8,814) = 7.98, *p* < 0.01] on GHQ-12 summary score in healthy controls. Similarly, there was a main effect of age [*F*(1, 8,814) = 58.47, *p* < 0.001], monthly income [*F*(1, 8,814) = 16.42, *p* < 0.001], highest educational qualification [*F*(1, 8,814) = 6.41, *p* < 0.01], legal marital status [*F*(1, 8,814) = 19.73, *p* < 0.001] and residence [*F*(1, 8,814) = 6.51, *p* < 0.05] on GHQ-12A (social dysfunction & anhedonia). However, the main effect of sex was insignificant. Moreover, there was a main effect of age [*F*(1, 8,814) = 120.38, *p* < 0.001], sex [*F*(1, 8,814) = 69.70, *p* < 0.001], monthly income [*F*(1, 8,814) = 6.38, *p* < 0.05], highest educational qualification [*F*(1, 8,814) = 5.54, *p* < 0.05], legal marital status [*F*(1, 8,814) = 11.87, *p* < 0.001] and residence [*F*(1, 8,814) = 10.96, *p* < 0.001] on GHQ-12B (depression & anxiety). Finally, there was a main effect of monthly income [*F*(1, 8,814) = 12.47, *p* < 0.001], highest educational qualification [*F*(1, 8,814) = 12.07, *p* < 0.001], and legal marital status [*F*(1, 8,814) = 75.91, *p* < 0.001]. However, the main effect of age, sex, and residence was not significant.

**Figure 1:**
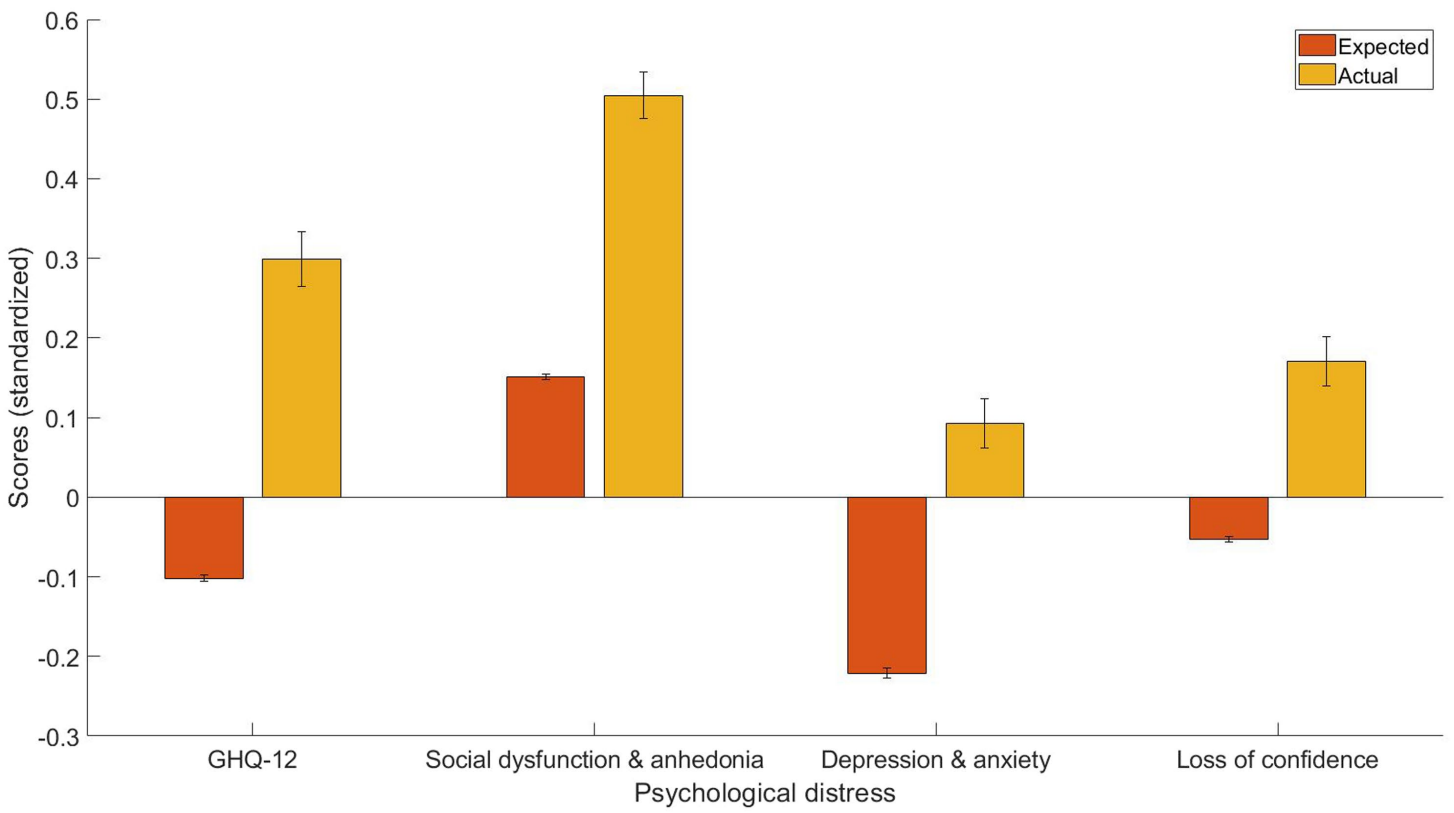
The expected and predicted GHQ-12 summary scores, GHQ-12A (social dysfunction & anhedonia), GHQ-12B (depression & anxiety), and GHQ-12C (loss of confidence) for people with angina.

Additionally, it was observed that individuals with angina had a significantly higher level of psychological distress, based on the GHQ-12 summary score [*t*(1080) = 11.75, *p* < 0.001, Cohen’s *d* = 0.40, 95% C.I. (0.33, 0.47)], GHQ-12A [*t*(1080) = 12.01, *p* < 0.001, Cohen’s *d* = 0.35, 95% C.I. (0.30, 0.41)], GHQ-12B [*t*(1080) = 10.57, *p* < 0.001, Cohen’s *d* = 0.31, 95% C.I. (0.26, 0.37)], GHQ-12C [*t*(1080) = 7.37, *p* < 0.001, Cohen’s *d* = 0.22, 95% C.I. (0.16, 0.28)]. Further, Figure 1 provides a visual representation of the mean and standard error of predicted and actual standardized scores.

## 4. Discussion

The aim of this research was to examine the influence of angina on individuals’ psychological distress, including its impact on three distinct dimensions of psychological distress, namely GHQ-12A (comprising social dysfunction and anhedonia), GHQ-12B (encompassing depression and anxiety), and GHQ-12C (reflecting loss of confidence). Using a predictive normative modeling approach with one-sample *t*-tests, a total of 1,081 participants with angina and 8,821 without angina were examined through the UKHLS dataset. Results revealed that individuals with angina experience greater levels of psychological distress compared to those without angina, as demonstrated by higher GHQ-12 summary scores (Cohen’s *d* = 0.31), GHQ-12A (Cohen’s *d* = 0.34), GHQ-12B (Cohen’s *d* = 0.21), and GHQ-12C (Cohen’s *d* = 0.20). To the best of my knowledge, this study is the first one that demonstrated the feasibility of using the GHQ-12 to screen psychological distress in individuals with angina and looked at how dimensions of psychological distress are affected by angina.

In this study, a CFA was conducted to identify underlying factors in the GHQ-12 scale. The analysis revealed a three-factor solution, including GHQ-12A (consisting of 6 items related to social dysfunction and anhedonia), GHQ-12B (consisting of 4 items related to depression and anxiety), and GHQ-12C (consisting of 2 items related to loss of confidence). The results of this study are consistent with previous research studies which also identified three factors in the GHQ-12 scale, as reported in studies by Campbell and Knowles (8), El-Metwally et al. (6), Gao et al. (7), Graetz (9), Martin et al. (10), Padrón et al. (11), Penninkilampi-Kerola et al. (12), and Rajabi et al. (13). Furthermore, the factor loadings in the current study were found to be high, as presented in Table 2.

The results showed that participants with angina have higher psychological distress is also consistent with previous studies [e.g., (1, 21, 27–32)]. Moreover, it has been demonstrated that the presence of new or persistent psychological distress can considerably heighten the likelihood of a future diagnosis of angina, as evidenced by follow-up research (32). Moreover, a large-scale study discovered that there are positive associations between psychological distress and self-reported clinical diagnoses of angina or heart attack (21). Another more recent study found that angina is associated with more psychological distress (1). The finding that participants with angina have more social dysfunction & anhedonia is consistent with studies that found a positive association between cardiovascular disease and social dysfunction & anhedonia [e.g., (35)]. Moreover, the effect size as indicated by Cohen’s *d* was the biggest for social dysfunction & anhedonia, which may indicate that dysfunction & anhedonia is the most pronounced psychological distress problem in people with angina but was largely ignored in the literature. The finding that participants with angina have more depression & anxiety problems is also consistent with several studies [e.g., (28, 30)]. Finally, participants with angina had more loss of confidence problems, which is of particular importance given that confidence in patients is closely related to clinical outcomes. Furthermore, the varying effect size of distinct dimensions of psychological distress suggests that the impact of angina on different dimensions of psychological distress may differ. This implies that an examination of specific dimensions of psychological distress is warranted in addition to assessing general psychological distress.

Angina has been found to be linked to psychological distress through various pathways. Psychological distress has been found to have a significant impact on the risk of myocardial ischemic changes, as observed through exercise electrocardiography or nuclear medical imaging examinations, as well as on the frequency of angina in patients with acute myocardial infarction. This is attributed to a combination of behavioral and biological factors, with negative emotions experienced during psychological distress leading to a decrease in medical compliance and health-promoting behaviors. Moreover, psychological distress can affect various biological activities, including those of the hypothalamic-pituitary-adrenal axis, autonomic nerves, platelets, and inflammatory cytokines, potentially increasing the risk of coronary artery disease such as angina. These factors can induce endothelial dysfunction and atherosclerosis, cause vasoconstriction, accelerate heartbeat, increase ventricular load, and raise the incidence of angina symptoms. The association between psychological distress and angina is also influenced by the interactions between behavioral and biological factors. For example, non-adherence to antidepressant medications and physical inactivity can increase inflammation and depressive symptoms in individuals with angina.

Although the current study has several strengths, it also has some limitations that need to be acknowledged. Firstly, the study’s cross-sectional design hinders the ability to establish causality due to the possible bidirectional association between psychological distress and angina. Longitudinal studies would be useful in determining the causal relationship between these two variables. Secondly, angina was evaluated using self-reported responses, thus the study cannot confirm the association between angina and clinical medical evidence or diagnoses. Hence, the current study does not establish whether angina was caused by cardiac or noncardiac factors. Future research could investigate how different types of angina, grouped by their causes, may relate to psychological distress differently. The study’s exclusive focus on individuals with angina in the United Kingdom presents a challenge in generalizing the findings to other cultural or national contexts. As a result, it is imperative that future research endeavors aim to reproduce the current results in diverse settings.

The current study aimed to investigate the impact of angina on general and dimensional aspects of psychological distress. The results indicated that psychological distress, both general and dimensional, was affected by angina. These findings support the validity of using GHQ-12 as a measure of psychological distress in individuals with angina. Moreover, the study suggests that it is crucial to consider all dimensions of psychological distress rather than solely focusing on specific dimensions, such as depression or anxiety, in individuals with angina. Clinicians should come up with interventions to reduce psychological distress in people with angina, which can then lead to better outcomes.

## Data availability statement

Publicly available datasets were analyzed in this study. This data can be found at: https://www.understandingsociety.ac.uk.

## Ethics statement

The studies involving human participants were reviewed and approved by University of Essex. The patients/participants provided their written informed consent to participate in this study.

## Author contributions

WK: conceptualization, data curation, formal analysis, investigation, methodology, resources, software, writing—original draft, and writing—review and editing.

## Funding

This work was supported by the Imperial Open Access Fund.

## Conflict of interest

The author declares that the research was conducted in the absence of any commercial or financial relationships that could be construed as a potential conflict of interest.

## Publisher’s note

All claims expressed in this article are solely those of the authors and do not necessarily represent those of their affiliated organizations, or those of the publisher, the editors and the reviewers. Any product that may be evaluated in this article, or claim that may be made by its manufacturer, is not guaranteed or endorsed by the publisher.
